# Structural consequences of arrested foveal development in preterms with persisting signs of immaturity

**DOI:** 10.1038/s41433-019-0627-4

**Published:** 2019-10-23

**Authors:** Johan Sjöstrand, Zoran Popović

**Affiliations:** 1 Section of Ophthalmology, Department of Clinical Neuroscience, Institute of Neuroscience and Physiology, Sahlgrenska Academy, University of Gothenburg, Gothenburg, Sweden; 2 Department of Ophthalmology, Sahlgrenska University Hospital, Gothenburg, Region Västra Götaland Sweden

**Keywords:** Retina, Eye manifestations

## Abstract

**Purpose:**

To evaluate the impact of structural changes in a limited sample of adult preterms with foveal immaturity from optical coherence tomography (OCT) B-scan images and to estimate layer displacement and changes in areal and volume magnification within the inner fovea.

**Subjects and methods:**

Layer thickness was measured in conventional and directional OCT scans from eight preterms with different degrees of foveal immaturity (24–33 weeks of gestation, 22–33 years of age) and five controls (20–33 years of age). We obtained reflectivity profiles of the outer plexiform layer (OPL) and manual segmentation data of the inner nuclear layer (INL) and the combined ganglion cell layer (GCL) and inner plexiform layer (IPL) at specified eccentricities from 300 to 900 µm. Displacement of cumulative thickness curves of preterms compared with that of the controls was used to estimate retardation of layer displacement. Changes in areal magnification and layer thickness were used to construct a structural model of redistribution within the fovea of preterms.

**Results:**

Retardation of centrifugal layer displacement of OPL and all inner retinal layers (IRL) was marked in both preterm groups with foveal immaturity, whereas retardation was marginal in the preterm group without clinical signs of immaturity. Retarded displacement within the IRL and OPL had a major impact on available space within the central fovea.

**Conclusions:**

A marked retardation of displacement was demonstrated for all IRL within the immature fovea of preterms with decreased areal and volume magnification and reduced space available for synaptic communication coupled to the degree of immaturity.

## Introduction

Marked changes occur in the human fovea during normal development. Histological studies show that towards the latter part of gestation the foveal pit deepens and that neurons within the ganglion cell layer (GCL) and inner nuclear layer (INL) start to disappear from the foveal centre (FC) [1–3]. The maturation continues postnatally and the disappearance of inner retinal layers (IRL) cells and cone pedicles is not complete until 17th months after term birth [4]. High resolution optical coherence tomography (OCT) studies have confirmed these maturation changes around birth [5, 6] and also demonstrated persistent signs of immaturity in preterms both with and without retinopathy of prematurity (ROP) [7–12]. In general, these studies have found that the maturation and lengthening of inner segments (IS) and outer segments (OS) of the foveal photoreceptors is within normal range.

Characteristic persisting signs of immaturity in preterms are increased thickness of the different layers of the inner retina together with presence of a sheet of outer plexiform layer (OPL) covering FC in combination with a reduced foveal depression [11]. An increased IRL thickness associated with a decreased foveal depth (FD) is still observed at adult age in most preterms born before 28 weeks of gestation [10, 13]. This lack of extrusion of the inner part of the fovea has been explained as an arrest of normal maturation. Since normal foveal development of the inner retina has been proposed to involve centrifugal migration of cells out from FC before and after birth [14], it is probable that an arrested extrusion of IRL from FC could be caused by a retarded centrifugal displacement of inner cell structures.

We compared thickness profiles of IRL and OPL to estimate the displacement difference of various layers in preterms versus controls. Our aim was to analyse the proposal of retarded displacement in preterms. Furthermore, the purpose was to evaluate the structural consequences of immaturity for cells and networks in terms of available space. We considered an estimate of possible retardation of IRL displacement from FC to be of great interest to evaluate possible functional impact of immaturity.

It has been estimated that the available space of foveal cone pedicles is increased due to the effect of areal magnification caused by lateral displacement during normal development in monkeys [15, 16]. A structural model based on changes in areal magnification [16] was established in order to evaluate the structural consequences of the changed displacement of different IRL structures in preterms with mild/moderate or severe signs of foveal immaturity.

## Methods

### Subjects and image acquisition

A detailed description of the subjects and the imaging procedure is given elsewhere [11] but is outlined here.

Selected eyes from eight young adults with a history of prematurity (24–33 weeks of gestation, 22–33 years of age at examination) and five controls (born at term, 20–33 years of age at examination) were imaged using conventional and directional SD-OCT with a Cirrus HD-OCT (Carl Zeiss Meditec, Inc., Dublin, CA, USA). The preterms were divided into three prematurity groups (PG) after clinical grading of OCT scans based on the signs of reduced FD and the presence of post-receptor layers or IRL at FC as follows: PG1 (*n* = 3) - no clinical signs of foveal immaturity and no ROP, PG2 (*n* = 3) - incomplete IRL displacement with a shallow foveal pit and no ROP, and PG3 (*n* = 2) - incomplete IRL displacement with very shallow foveal pit and treated ROP. Inclusion criteria for controls (*n* = 5) were birth at term, no history of ocular disease and absence of visual complaint.

The HD 5 Line Raster protocol without spacing between scans and 20 scans per B-scan was used to capture high-definition images. Retinal layer thickness analysis of directional SD-OCT images was performed at selected temporal eccentricities defined by the individual distance between FC and the foveal rim (R) for each case. Images were registered, averaged and flattened to the posterior retinal pigment epithelium boundary using a semiautomatic custom software written in MATLAB (2014a; Mathworks, Natick, MA, USA).

The temporal side of the SD-OCT B-scan images was selected for analysis to avoid the asymmetry caused by the marked thickening of the retinal nerve fibre layer on the nasal side. Measurements were performed at selected temporal eccentricities defined by the individual distance between FC and the foveal rim for each case. Individual retinal scaling factors were calculated from measurements of axial length to compensate for differences in lateral magnification in all cases except the PG3 group [17]. Layer thickness was measured in straight scans, i.e. horizontal SD-OCT B-scans with entrance beam positions through the pupil centre with a distinct light reflex in the foveal pit, and tilt-up scans, obtained with OCT entrance beam positions displaced from the pupil centre, from preterms and controls as described in a previous study [11]. We obtained reflectivity profiles of the OPL and manual segmentation of the INL, the combined GCL and inner plexiform layer (IPL), and the combined Henle fibre layer (HFL) and outer nuclear layer (ONL). Layer thickness in reflectivity profiles was defined as the thickness at the midway point between peak reflectivity of the hyperreflective layer and the trough reflectivity of the hyporeflective outer border of INL. A thickness ratio (PG/C) was calculated for each layer at selected eccentricities (Table 1).

**Table 1:** Retardation and PG/C thickness, area and volume ratios at selected control group eccentricities

	Eccentricity	Retardation			PG/C thickness ratio			PG/C area ratio			PG/C volume ratio		
	C	PG1	PG2	PG3	PG1	PG2	PG3	PG1	PG2	PG3	PG1	PG2	PG3
GCL + IPL	300	31	113	252	1.19	0.84	2.19	0.80	0.39	0.03	0.96	0.33	0.06
	500	48	120	313	0.83	0.90	0.94	0.82	0.58	0.14	0.68	0.52	0.13
	700	54	78	288	0.83	0.93	1.04	0.85	0.79	0.35	0.70	0.74	0.36
	900	61	50	264	0.80	0.86	1.11	0.87	0.89	0.50	0.70	0.77	0.55
INL	300	13	185	250	1.01	1.04	2.03	0.91	0.15	0.03	0.92	0.15	0.06
	500	10	171	315	1.20	1.13	1.44	0.96	0.43	0.14	1.15	0.49	0.20
	700	40	189	363	1.05	1.08	1.20	0.89	0.53	0.23	0.93	0.58	0.28
	900	70	222	412	0.96	0.98	1.05	0.85	0.57	0.29	0.81	0.56	0.31
OPL	300	27	156	199	1.20	0.77	1.14	0.83	0.23	0.11	1.00	0.18	0.13
	500	58	146	262	1.25	1.12	1.26	0.78	0.50	0.23	0.98	0.56	0.28
	700	65	151	279	1.03	1.01	1.05	0.82	0.62	0.36	0.85	0.62	0.38
	900	72	155	295	0.88	0.91	0.91	0.85	0.68	0.45	0.74	0.63	0.41

		C			PG1			PG2			PG3		
RT @ FC		185 ± 10			180 ± 27			218 ± 19			322 ± 13		
FD @ FC		121 ± 15			119 ± 6			78 ± 38			12 ± 20		
IRLt @ FC		8 ± 2			5 ± 1			23 ± 12			84 ± 5		
FDA @ FC		13 ± 3			21 ± 4			5 ± 2			2 ± 0		

*GCL* ganglion cell layer, *IPL* inner plexiform layer, *INL* inner nuclear layer, *OPL* outer plexiform layer, *FC* foveal centre, *RT* retinal thickness, *FD* foveal depth, *IRLt* IRL thickness, *FDA* foveal developmental arrest (HFLt + ONLt)/(IRLt + OPLt)

### Data analysis

**Fig. 1:**
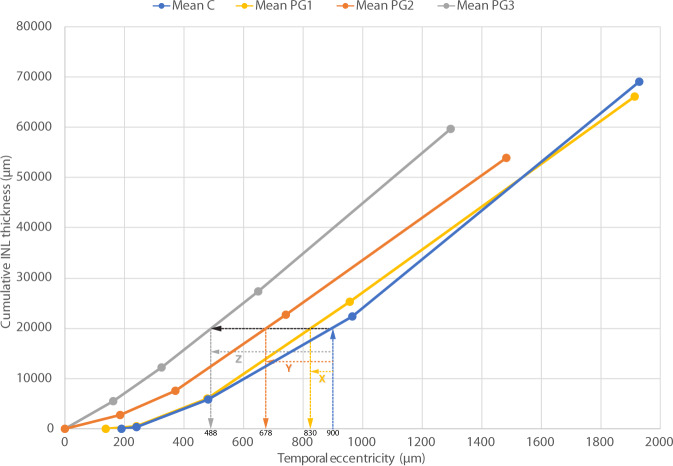
Cumulative INL layer thickness as a function of temporal eccentricity. Retardation from a specified temporal eccentricity in controls is obtained by selecting a location along the eccentricity axis, moving vertically to intersect the cumulative function of controls (C), and measuring the horizontal distance (*X* for PG1, *Y* for PG2 and *Z* for PG3) to the corresponding cumulative function (c.f. Packer et al. [18]). An example for the case of INL retardation at a C eccentricity of 900 µm is shown by the dashed lines (c.f. Table 1)

Thickness profiles of IRL and OPL were compared to estimate the displacement difference of various layers in PG versus controls. The displacement of cumulative thickness curves of preterms from that of controls were used to estimate retardation of layer displacement. The area under the curve was calculated using proFit v6.2.16 (Quantum Soft, Uetikon am See, Switzerland) as the definite integral of a spline curve fit to the data points of mean IRL thickness, mean HFL + ONL thickness and mean OPL thickness of C and PG. The lower and upper integral limits for the cumulative curves were values from a minimum eccentricity of zero to a maximum eccentricity defined by selected landmarks. Retardation from a specified temporal eccentricity in controls was obtained by selecting a location along the eccentricity axis, moving vertically to intersect the cumulative function of controls, and measuring the horizontal distance to the corresponding cumulative function (c.f. Packer et al. [18] and Fig. 1).

A foveal development arrest (FDA) index was calculated to relate retardation to severity of immaturity and obtain a quantifiable measure of immaturity at FC. Our FDA index, which is a modification of the macular developmental arrest (MDA) index described by Bowl et al. [19], was defined as the thickness ratio of HFL + ONL and the sum of IRL and OPL (Table 1) at FC.

A structural model based on the areal magnification [16] was established in order to evaluate the structural consequences of the changed displacement of different IRL structures in preterms with mild/moderate or severe signs of foveal immaturity.

The research followed the tenets of the Declaration of Helsinki. All subjects were informed about the goals, consequences and protocol of the study, and then provided their written informed consent.

## Results

### Layer displacement changes of IRL and OPL in preterms

**Fig. 2:**
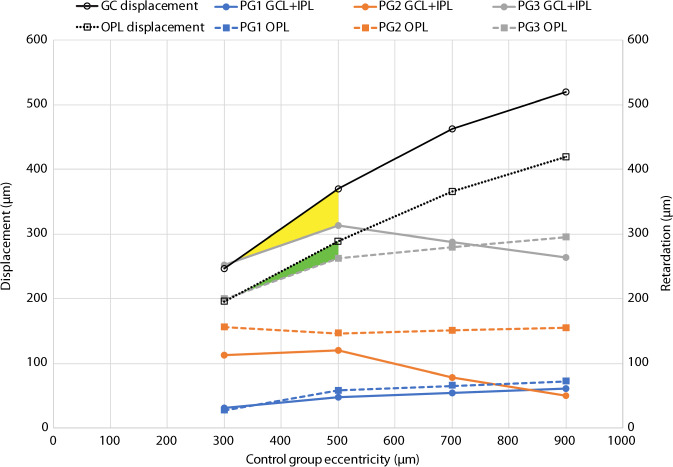
Retardation of GCL + IPL and OPL as a function of control group eccentricity. The black continuous line shows normal GC displacement and the black dashed line shows normal OPL displacement from Drasdo et al. [20]. The estimated residual displacement of GCL + IPL and OPL in PG3 within the central fovea is represented by the yellow and green shaded areas, respectively

The centrifugal displacement of all inner foveal layers (GCL + IPL, INL and OPL) from FC towards the foveal rim were retarded compared with controls in all PG (Table 1). Group averages of retardation in preterms calculated from the horizontal displacement of cumulative thickness curves of inner foveal layers in preterms compared with controls varied between the different PG (Fig. 1). The most marked retardation was observed in the group with severe signs of prematurity (PG3) and least in preterms without clinical signs of immaturity (PG1). The FDA index expressed as a ratio of the HFL + ONL thickness and IRL + OPL thickness (Table 1) was related to the magnitude of retardation, with a mean ratio of ≤5 in both groups with foveal immaturity (PG2 and PG3). Different layers within each group of preterms generally showed limited changes at different eccentricities (Fig. 2).

**Fig. 3:**
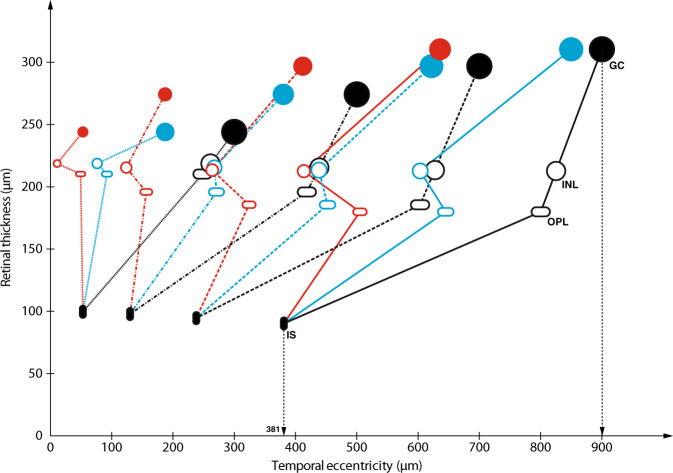
A model of the structural consequences of a graded retardation of displacement of GCL + IPL, INL and OPL. Cone inner segment (IS) positions are assumed to be constant when comparing C with PG2 and PG3 at the selected control group eccentricities. An example of the corresponding positions of IS and GC at 900 µm eccentricity according to the data from Drasdo et al. [20] is shown by the vertical dotted lines. Symbol sizes of GC, INL and OPL of controls are kept constant at different eccentricities in order to facilitate comparisons of size reduction in PG2 and PG3. Structural size and area are minified in retarded layers due to the lower areal magnification caused by reduced layer displacement from the foveal centre. C—black lines; PG2—blue lines; PG3—red lines; GC—filled circles; INL—open circles; OPL—open ellipse; IS—filled ellipse

The average retardation of all layers was marked within the foveal region (300–900 µm eccentricity) in PG2 and PG3, i.e. groups with IRL persistence and reduced FD (Table 1), whereas retardation was marginal (mean 46 µm, range 10–72 µm) in PG1, i.e. the preterm group with normal FD and IRL extruded from FC. Average retardation within the foveal region in the two groups with structural signs of immaturity (PG2 and PG3) was 145 µm (range 50–222 µm) and 291 µm (range 199–412 µm), respectively. Some difference in retardation was seen between layers and position within the fovea (Figs. 2 and 3). In general, retardation was most marked for INL.

Layer displacements of PG2 and PG3 compared with reported values of displacement of ganglion cells and cone pedicles from corresponding cones in normal fovea [20] are presented in Figs. 2 and 3. The retardation in the preterm groups relative to normal displacement from cones gives an estimate of the magnitude of the blockage of displacement in each layer. The blockage is most marked for all layers within the central part of the fovea in PG3. The percentage of retardation compared with normal decreased from the central to the peripheral part of the fovea for most layers as can be seen in Fig. 2. Average foveal retardation as a percentage of normal displacement for PG2 and PG3 was ~51% and 86% for INL and 26% and 75% for GCL + IPL, respectively.

### Area/volume changes due to retarded migration

**Fig. 4:**
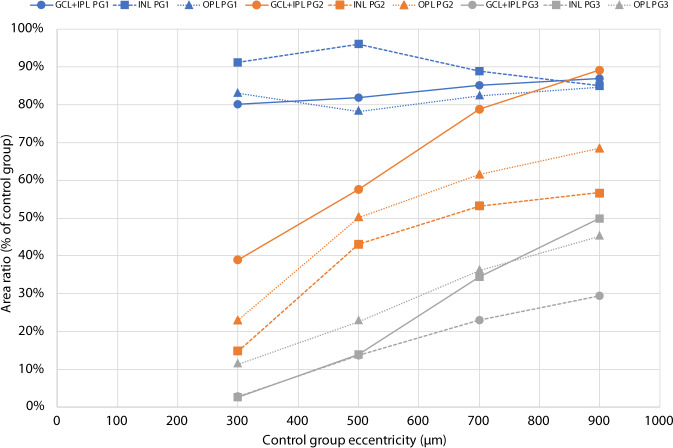
Areal ratios as a function of control group eccentricity for GCL + IPL, INL and OPL of PG compared with controls at corresponding eccentricities (c.f. Fig. 3)

A model of the structural consequences of a graded retardation of displacement of IRL and OPL was constructed using the parameters retardation, areal magnification and layer thickness ratio (Table 1 and Fig. 3). The size and area of structures are minified in retarded layers due to the lower areal magnification caused by the decreased displacement of layers away from FC compared with controls. This areal minification was most marked centrally (Table 1 and Fig. 4). Assuming a constant number of cell structures in preterms and controls implies a proportional change in density and size. An estimate of the size reduction necessary to accommodate the same number of cell structures within a decreased available area may be calculated using the areal ratio of OPL in preterms (Table 1 and Fig. 4) and the reported cone pedicle density of 25,000 cells/mm^2^ in normals [21]. The average normal pedicle diameter of 6.3 µm has to be reduced to 3.0 µm and 2.1 µm at 300 µm eccentricity and 4.5 µm and 3.0 µm at 500 µm eccentricity for PG2 and PG3, respectively.

The reduction of volume or available space compared with that of the controls was most marked in the different layers of the central part of the fovea and the average reduction within the foveal region ranged from 37 to 62% and from 21 to 40% of that of controls in preterms with mild/moderate immaturity (PG2) and with severe signs (PG3), respectively (Table 1).

The maximal volume decrease for all layers within the immature retina was found at the most central eccentricity (300 µm) where it ranged from 15 to 33% in PG2 and 6 to 13% in PG3. An increased thickness ratio (Table 1) modifies the volume change for GCL + IPL and INL in the group with severe immaturity (PG3). Modelling the volume change in the OPL layer as the change in volume of a sphere at 300 µm eccentricity corresponds to a diameter of 61% and 48% of that of the controls for PG2 and PG3, respectively (Fig. 3).

## Discussion

The major finding of the present follow-up study is that an immature fovea in preterms is associated with a marked retardation of centrifugal displacement of IRL and OPL compared with controls. Although our results cannot be generalised due to the low number of subjects, our model confirms the proposal of an arrested migration of inner retinal cells and cone pedicles in preterms with signs of foveal immaturity during foveal development [10, 11, 22].

The degree and extent of retardation in each group of preterms seems related to the severity of signs of immaturity characterising the group as shown by the marked difference in retardation among the three PG selected on the basis of degree of IRL persistence and FD. A calculation of an FDA index confirmed that the magnitude of retardation was related to this index and thus to a quantifiable measure of immaturity at FC. Our FDA index (Table 1) is a modified version of the MDA index described by Bowl et al. [19]. In an agreement with this study we found that such an index is useful to estimate foveal developmental arrest since no overlap of the ratio was found between the cases with (PG2 and PG3) and without (PG1 and controls) clinical signs of immaturity.

Comparison of profiles of cumulative layer thickness curves allowed us to calculate the average horizontal displacement, i.e. retardation, of the GCL + IPL, INL and OPL in preterms. This calculation was dependent on the precision of the segmentation and delineation of the inner and outer borders of each layer. Manual segmentation of GCL + IPL and INL was shown to be comparable with reflectivity profile thickness measurements in our previous study [11]. We therefore used reflectivity profiles of tilted OCT images to define of the borders of the OPL that show a better correlation to histological measurements of OPL [23].

The magnitude of retardation showed some variation between different layers and positions and was generally most marked for INL and least for GCL + IPL in PG2. A possible explanation for this difference in measured displacement could be that part of the GCL/IPL extrusion from FC has already occurred during normal foveal development [24] before the start of developmental arrest is induced by the premature birth. Evidence in line with this explanation has been presented in a longitudinal OCT study of preterms indicating that the extrusion of IRL is sequential with GCL/IPL disappearing before INL during the early phase after premature birth [25]. Another explanation for the difference in retardation within each immaturity group could be that the horizontal traction forces postulated to act during normal development [22] are differently blocked to some extent.

A comparison of the observed retardation of different layers at various eccentricities to published histological data of normal displacement of ganglion cells and cone pedicles [20] reveals that the blockage of displacement of all layers is almost total with a residual of 10% or less within the central part of fovea in PG3. Residual displacement in PG2 is approximately one-third for INL and OPL and two-thirds for GCL + IPL compared with estimated total normal displacement centrally.

Foveal development involves two distinctive displacement processes taking place in both the outer and inner retina, namely the centrifugal displacement of GCL and INL cells to the foveal rim and the centripetal displacement and elongation of photoreceptors towards FC [26]. The residual displacement compared with that of the control within the peripheral part of the primate fovea was ~40% for GCL + IPL and OPL in the peripheral part of the fovea of PG3, whereas it was higher for all layers in PG2. However, some part of this residual displacement in the peripheral part may be caused by an unchanged centripetal displacement of photoreceptors in this region. A calculation based on the data of Packer et al. [18] of centripetal displacement of cones in monkeys in relation to published data of total displacement [16, 27] indicates that the centripetal displacement of cones in the peripheral part of the fovea may be 40–50% of total displacement between cone pedicles and IS in monkeys. If this is true in humans it would suggest that a residual of 40% or less in the peripheral part of the fovea, such as for PG3, is an indication of a total blockage of centrifugal displacement within all parts of the fovea in PG3 and of approximately two-thirds or more in PG2.

The structural consequence of a graded retardation of IRL and OPL in preterms with an immature fovea were modelled as a change of size and areal magnification [16]. Schein [16] demonstrated a marked difference in available space caused by areal magnification between foveolar IS and connected cone pedicles. The decreased size and areal magnification demonstrated in our model indicates that marked reductions of available area for pedicles, dendritic networks and axonal terminal arborizations of the inner retina occur due to retarded displacement of GCL/IPL, INL and OPL.

Our estimate of an average size reduction from a pedicle size of 6.3 µm in controls within the central fovea of ~40% for PG2 and 60% for PG3 indicates that the central pedicles in the immature fovea are approximately halved in size due to retarded displacement. A calculation of the disparity in available area for cone IS based on the magnitude of lateral displacement of cone pedicles [20] and its impact on areal magnification in normal adults yields an interesting comparison with the size effect resulting from completely blocked lateral displacement of cone pedicles. We estimate a 50% size reduction of central pedicles in cases with no displacement compared with normal using density values for cones [28, 29] and for pedicles [21] of normal adult human fovea. This indicates that central pedicle displacement in cases with immaturity is more or less completely blocked.

A possible functional correlate to the calculated structural minification of INL and OPL structures could be the attenuation of cone mediated multifocal ERG (mfERG) responses within the fovea observed in the macula of preterms with and without ROP in a number of recent studies [30–33]. Since the mfERG response in a cone-initiated activity mainly reflects bipolar cell activity [34] it is possible that the decreased space available for the synaptic networks within OPL and INL is related to the reduced mfERG signals. These mfERG reports presented limited changes of VA and lack of correlation between mfERG responses and VA. Visual dysfunction revealed as reduced light sensitivity with no correlation to VA has been described by Bowl et al. [35, 36] in a subgroup of preterms with immature foveal structure. However, other studies have shown a correlation between VA and foveal parameters such as IRL persistence and increased area of IRL centrally [37–39].

The structural consequences of minified cone pedicles and synaptic networks can play a critical role in more complex visual functions where all synaptic couplings need to be optimal. This may explain the discrepancy between the effect on different visual functions with lack of correlation between reduced visual responses and visual acuity. Electron microscopy and immunocytochemical studies in primates have revealed a complex cone pedicle architecture [40]. A marked minification of foveal pedicles from a normal size of 6.3 µm in the human foveal slope [21] could have an impact on complex central functions. It has been discussed that even in the normal primate fovea there may be limited space for synaptic contacts in the most central smaller cone pedicles located at the edge where the cone pedicles start to form a continuous layer [41]. The volume available for synaptic communication is still markedly reduced even though some elongation of structures may be possible due to an increased layer thickness ratio for some retarded IRL as shown in our study.

According to our opinion it is therefore too early to state that the foveal pit is of visual insignificance based on the fact that visual acuity and foveal cone inner and OS specialisation is within normal range [42] in spite of changed inner retinal morphology. In line with this a recent study of infants with ROP has shown that grading using a measure of MDA has a predictive value for VA and mfERG responses [19]. The probability that abnormal inner foveal structure may have functional consequences raises the question whether there is a time window of opportunity for intervention and remodelling of foveal architecture following an early diagnosis of immaturity after preterm birth [43].

There were limitations to this study. The low number of subjects may limit our conclusions regarding a retarded centrifugal displacement of IRL due to the low statistical power of our analyses. However, the use of characteristic landmarks of the fovea established in the previous study [11], the groups of preterms defined by the degree of immaturity, and the use of both straight and tilted OCT images through a well-defined FC, gives support for a generalised retardation of displacement within the immature inner retina.

Another limitation is the measurement and analysis of the OPL, a very thin layer with a thickness of 2–5 pixels. A layer thickness measurement difference of one pixel will thus result in large variations. Our observation was that measurements from straight images yielded unreasonable OPL thickness values when compared with data from the literature [23]. A better correlation was obtained through the use of reflectivity profiles from tilted directional OCT images.

In conclusion, our study of the immature fovea presents evidence for a retardation of normal centrifugal displacement of inner cell structures from FC and the foveal rim. A model of the structural consequences of foveal immaturity indicates that available space for synaptic communication is markedly reduced within the inner part of the central fovea due to the retarded displacement. Further studies of the structural impact of premature birth and foveal immaturity on central visual functions using larger populations of preterms are needed in order to confirm the findings of the present study and elucidate the consequences of arrested foveal development for vision.

## Summary

### What was known before

- Characteristic persisting signs of immaturity in preterms are an increased thickness of inner retinal layers in combination with a sheet of outer plexiform layer covering the foveal centre and a reduced foveal depression.
- An increased thickness of inner retinal layers associated with a decreased foveal depression is still observed at adult age in most preterms born before 28 weeks of gestation.

### What this study adds

- Our study presents evidence of a retarded centrifugal displacement of inner cell structures from the foveal centre in the premature fovea.
- We present a model of the structural consequences of foveal immaturity for evaluation of decreased available space in the immature fovea.
